# Advances in Imaging-based Diagnosis and Treatment Strategies for AIDS-Related Cerebral Toxoplasmosis

**DOI:** 10.2174/0115734056415877260128072819

**Published:** 2026-02-17

**Authors:** Zhiguang Si, Hong Yang, Wangpin Meng, Ran Yang, Yunqing Wan

**Affiliations:** 1 Department of Medical Imaging, Affiliated Dehong Hospital of Kunming Medical University, Yunnan, P.R. China; 2 Department of Infectious Diseases, Affiliated Dehong Hospital of Kunming Medical University, Yunnan, P.R. China; 3 Department of Surgery, Affiliated Dehong Hospital of Kunming Medical University, Yunnan, P.R. China

**Keywords:** Cerebral toxoplasmosis, *Toxoplasma gondii*, AIDS, Neuroimaging, Opportunistic infections, AIDS-related cerebral toxoplasmosis

## Abstract

Cerebral toxoplasmosis is one of the most common opportunistic infections among AIDS patients. Clinical and neuroimaging manifestations are diverse and non-specific, resulting in frequent delayed diagnosis and even misdiagnosis, leading to neurological impairment, coma, and death. In addition to clinical and serological examinations, multimodal neuroimaging is indispensable for early diagnosis and subsequent treatment evaluation. Indeed, functional magnetic resonance imaging technologies and positron emission tomography provide complementary information for early diagnosis and treatment, which can improve prognosis when combined with prevention strategies. Recent advances in vaccine development have provided new hope for the prevention of cerebral toxoplasmosis. This article reviews multimodal imaging evaluation strategies and other recent clinical advances for the prevention, diagnosis, and treatment of AIDS-related cerebral toxoplasmosis.

## 1 INTRODUCTION

*Toxoplasma gondii* is a parasitic protozoan that is endemic in the environment, and one-third of the world's population is thought to be infected with T. gondii [1, 2]. While most such infections are latent, reactivation may occur when immune function is compromised. Among AIDS patients, *T. gondii* is the main pathogen responsible for opportunistic infection of the brain, termed cerebral toxoplasmosis. Symptoms usually present in the late stages of AIDS when CD4^+^T lymphocyte count falls below 200/µL, and risk rises markedly when CD4^+^T lymphocyte count falls below 50/µL [3-5]. Cerebral toxoplasmosis is a common cause of morbidity and mortality among AIDS patients, and thus a major risk factor for poor prognosis [6-9]. Timely diagnosis and treatment are critical for improving survival, but symptoms and signs are heterogeneous and generally nonspecific, necessitating high clinical suspicion and broad diagnostic testing. Neuroimaging is the primary means of early differential diagnosis [10-12], and combining functional Magnetic Resonance Imaging (fMRI) with Positron Emission Tomography (PET) modalities is recommended,especiallyforatypicalcases. Despite these challenges, there has been substantial progress in the diagnosis and treatment of AIDS-related cerebral toxoplasmosis due to advances in imaging technology combined with more effective strategies for prevention of opportunistic infections and anti-HIV (anti-retroviral, ART) treatments. This article reviews recent progress in the multimodal imaging evaluation of AIDS-related cerebral toxoplasmosis, therapeutic and preventative strategies, and critical challenges for future research.

We have conducted a literature search of studies published in PubMed and CNKI before October 11^th^, 2025, based on four components: Cerebral toxoplasmosis, Toxoplasma gondii, AIDS, and Neuroimaging. This article reviews multimodal imaging evaluation strategies and other recent clinical advances for the prevention, diagnosis, and treatment of AIDS-related cerebral toxoplasmosis.

## 2 IMAGING EVALUATION

### 2.1 Diagnostic value of Computed Tomography

At diagnosis, AIDS-related cerebral toxoplasmosis usually manifests with multiple lesions on neuroimages, while a single lesion is relatively rare. These lesions often occur in the basal ganglia and the corticomedullary junction, but are infrequent in the cerebellum, brainstem, and ventricles. Plain Computed Tomography (CT) scans usually show multiple low-density lesions as well as a few high-density lesions with hemorrhage. On contrast-enhanced scans, these lesions show annular and nodular enhancement, a space-filling effect, and obvious surrounding edema. In some cases, a ‘target sign’ is observed, formed by a surrounding annular enhanced area and mural nodules [13, 14]. However, the sensitivity and specificity of plain CT manifestations are relatively poor due to low spatial resolution, often leading to missed diagnoses, whereas lesions detected by enhanced CT are clearer and more specific for cerebral toxoplasmosis. In addition to revealing multiple space-occupying lesions, CT is suitable for detecting bleeding and calcification within lesions, signs helpful for differential diag-nosis. Although not the preferred imaging modality, CT can be used as a screening option, especially for patients with cont-raindications to MRI, due to its fast imaging speed and low cost.

### 2.2 Diagnostic value of MRI

The MRI manifestations of AIDS-related cerebral toxoplasmosis are diverse and have no obvious specificity, which may reflect image acquisition at different stages of infection, necrosis, and cystic degeneration. Patients may exhibit multiple or single low-intensity signals on T1-Weighted Images (T1WI) and high-intensity signals on T2-Weighted Images (T2WI), with obvious signs of edema and space-occupying effects. Typical imaging findings are frequently multinodular, with annular enhancement in the basal ganglia and the frontal, parietal, and occipital lobes, accompanied by perifocal edema, but with less involvement of the temporal lobe, brainstem, and cerebellum [15]. An eccentric or concentric ‘target sign’ is also frequently observed, formed by a circular area alternating between peripheral enhancement, a middle low-signal area, innermost enhancement, and a peripheral high-signal enhancement edge with slightly eccentric nodules [16-18]. Multimodal MRI is advantageous over CT due to higher spatial resolution in soft tissues, the ability to distinguish different tissue types, and the absence of a blind spot, resulting in a higher detection rate.

**Fig. (1):**
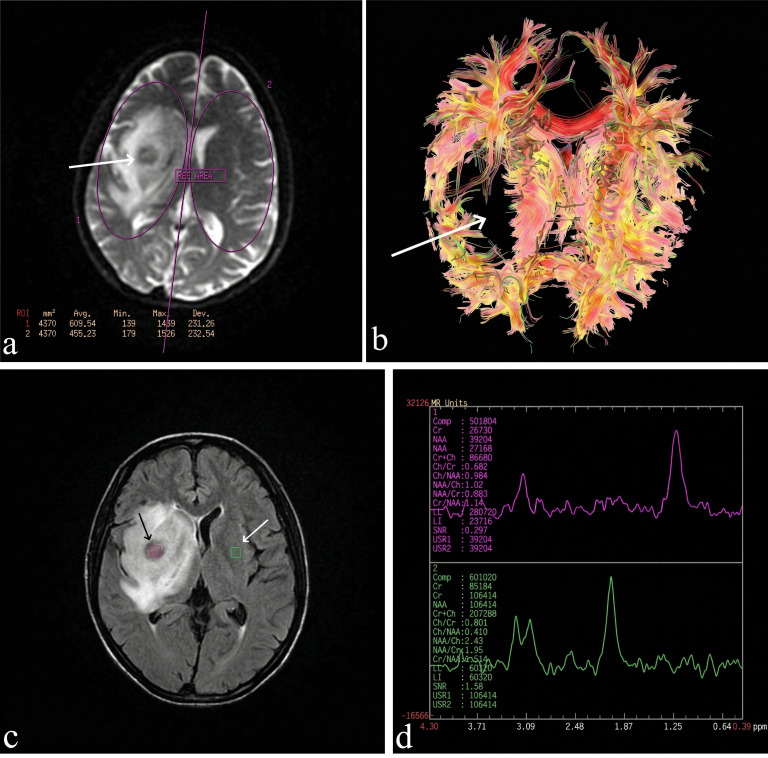
AIDS-related cerebral toxoplasmosis in a 38-year-old female. (**a**, **b**) Axial MRI T2WI and DTI images show lesions in the left basal ganglia area (arrow). (**c**, **d**) Axial MRI T2 fluid attenuated inversion recovery (T2 FLAIR) and MRS images show lesions in the right basal ganglia area (black arrow) and normal brain tissue in the left basal ganglia area (white arrow).

Functional MRI modalities, including Diffusion-Weighted Imaging (DWI), Diffusion Tensor Imaging (DTI), Perfusion-Weighted Imaging (PWI), and Magnetic Resonance Spectro-scopy (MRS), can also provide essential information for diagnosis. The manifestations of AIDS-related toxoplasmosis on DWI are diverse, with typical high-intensity signals in the center reflecting coagulation hemorrhagic necrosis. These necrotic areas contain mainly inflammatory cells, *T. gondii*, and proteins. Proteins strongly restrict the diffusion of water molecules, resulting in uneven high-intensity signals and low Apparent Diffusion Coefficient (ADC) values on DWI [19], while PWI can reveal reduced local Cerebral Blood Volume (CBV) around these *T. gondii-*induced lesions due to the lack of functioning blood vessels. In addition, DTI often reveals sparse fibrous bundles, some slightly compressed and displaced, suggesting damage to white matter tracts. Large and often fatal lesions also show reduced and displaced fibrous bundles around the lesion parenchyma (Fig. **1a, b**) [20]. Magnetic resonance spectroscopy can detect the levels of critical brain metabolites indicative of structural damage and metabolic disruption, such as N-Acetylaspartate (NAA), Creatine (CRE), Choline (CHO), Lipid (LIP), Lactate (LAC), and Myo-Inositol (MI). Gao *et al*. reported a significantly reduced CHO peak among AIDS-related cerebral toxoplasmosis patients, potentially reflecting inflammatory demyelination, proliferation of immune cells, and expansive gliosis. In addition, MRS revealed slightly reduced NAA indicative of neuronal damage, a reduced CRE peak related to energy failure and ischemia caused by rapid non-neuronal cell proliferation, a reduced MI peak reflecting reactive glial cell proliferation, and the appearance of LAC and LIP peaks suggestive of necrosis in the lesion parenchyma (Fig. **1c, d**) [21]. These functional MRI modalities thus provide complementary information on lesion size, location, and composition, damage to grey and white matter, and metabolic changes for the differential diagnosis of AIDS-related toxoplasmosis encephalopathy.

### 2.3 Diagnostic value of PET

**Fig. (2):**
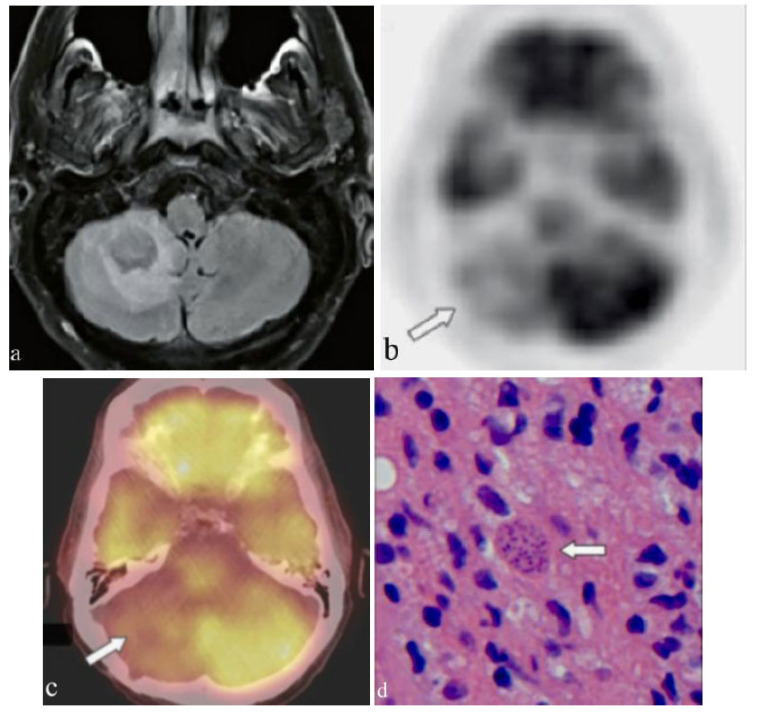
AIDS-related cerebral toxoplasmosis in a 61-year-old male. (**a**) Axial MRI T2 FLAIR image shows lesions in the right cerebellum. (**b**, **c**) Axial FDG PET and fused FDG PET/CT images show a significant decrease in metabolic activity in the right cerebellar hemisphere compared to the contralateral hemisphere (arrow). (**d**) Cerebellar lesion biopsy shows Toxoplasma gondii bradyzoites (arrow).

**Fig. (3):**
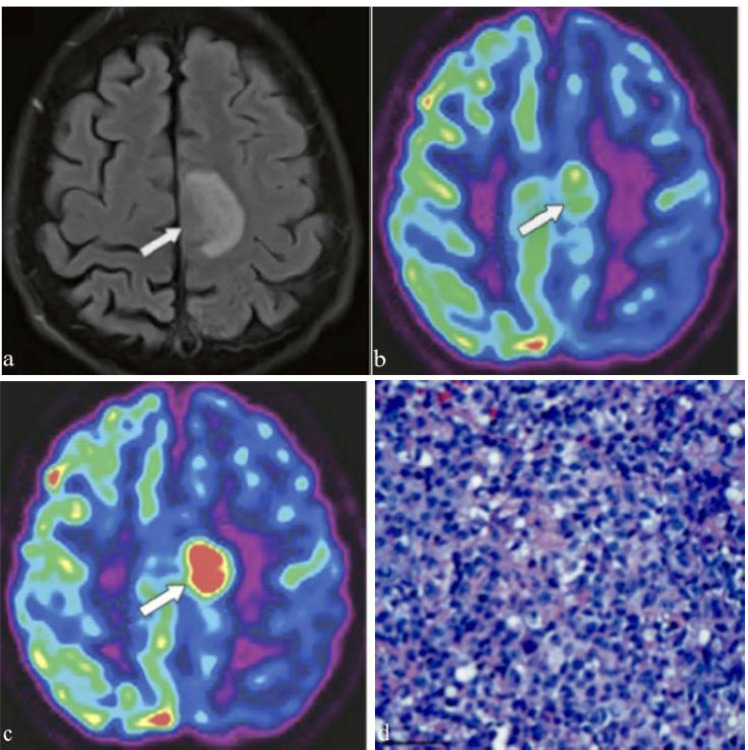
AIDS-related lymphoma in a 55-year-old male. (**a**) Axial MRI T2 FLAIR image shows a lesion in the posterior center of the left frontal lobe semioval (arrow). (**b**, **c**) Axial 1-hour and 5-hour FDG-PET/CT images show a progressive increase in metabolism at the center of the left semiovale over time (arrow). (**d**) Brain lesion biopsy shows invasive lesions composed of malignant lymphoid cells.

FDG-PET/CT is also used to identify AIDS-related cerebral toxoplasmosis and central nervous system lymphoma. Several studies have shown that the standard uptake value (SUV) of metabolic tracers (*e.g.*, 18F-fluorodeoxyglucose, FDG) is significantly lower in cerebral toxoplasmosis lesions than in nervous system lymphoma (Fig. **2a**-**d**) [22, 23]. Thus, delayed PET imaging allowing for substantial trace accumulation is considered a reliable method to distinguish infection from malignant tumors. Compared to normal tissue, malignant tumors accumulate substantially more tracer over time (Fig. **3a-d**) [24]. Further, the metabolic activity of benign lesions is lower than that of malignant lesions [25]. In a series of HIV-infected patients, Skiest *et al*. confirmed that Thallium-201 SPECT was able to accurately differentiate primary brain lymphoma from other causes of focal central nervous system lesions in most patients; however, both false positive and false negative results occurred [26, 27]. When FDG-PET/CT shows atypical manifestations in suspected cerebral toxoplasmosis cases, delayed imaging a few hours after administering the metabolic tracer in combination with other conventional imaging modalities can improve diagnostic accuracy by providing better contrast with the background. However, the cost and time required may limit the clinical application of these methods.

### 2.4 Multimodal Evaluation

Each of these modalities (CT, MRI, and PET) presents unique imaging manifestations for the diagnosis of AIDS-related cerebral toxoplasmosis. Although lesions can usually be detected on plain CT scans, missed detection is possible due to low spatial resolution. MRI sequences can achieve higher tissue resolution and further detect surrounding tissue edema, which is helpful for determining lesion scope and severity. Thus, conventional structural MRI sequences can detect AIDS-related cerebral toxoplasmosis lesions in most cases. When lesions are atypical, multimodal fMRI can provide important supplementary information for differential diagnosis [28]. Similarly, PET is mainly used for atypical cases, especially for the differentiation of AIDS-related cerebral toxoplasmosis from central nervous system lymphoma. Delayed PET imaging can further improve the accuracy of diagnosis, as rates of metabolic tracer uptake are distinct. Therefore, the combination of these imaging techniques can achieve high diagnostic accuracy and sensitive treatment efficacy evaluation. For patients with suspected cerebral toxoplasmosis, CT is used routinely to detect the lesions, and MRI to determine the nature and progression of the lesions (*i.e.*, extent of damage). When encountering atypical cases, the application of fMRI or PET examination is effective for differential diagnosis. Further research is essential to identify imaging signs with greater specificity and sensitivity for the detection of these lesions as early as possible for timely treatment initiation.

## 3 CLINICAL PROGRESS ON CEREBRAL TOXOPLAMOSIS DIAGNOSIS AND TREATMENT

### 3.1 Clinical Features

Like imaging manifestations, the symptoms of AIDS-related cerebral toxoplasmosis are heterogeneous, paralleling the variation in lesion location and number. Clinical symptoms include fever, headache, sensory or motor function changes, focal neurological deficits, disorientation, disturbance of consciousness, decreased consciousness, and seizures [29, 30]. Cerebral toxoplasmosis is rarely the first manifestation of AIDS, but incidence is very high in the later phases of the disease, and life-threatening if not treated on time [22, 31, 32]. Most *T. gondii* infections are dormant in the host, and the most common latent infection site is the central nervous system [33-35]. Histologically, infection manifests as diffuse encephalitis, cystic lesions, microglial nodules, and lymphocytic vasculitis. Infection occurs through a variety of routes, including ingestion of contaminated water or food, oral transmission through contact with cat litter containing *T. gondii* cysts, transmission through blood transfusion or organ transplantation, and mother-to-child transmission. When the immune system is damaged by AIDS or suppressed by drugs, cerebral toxoplasmosis may arise from reactivation of latent *T. gondii* infection [36].

A presumptive diagnosis of cerebral toxoplasmosis is usually possible based on the integration of clinical features, imaging features, and laboratory test results, especially when the CD4^+^T lymphocyte count is less than 200/µL. Indeed, if patients meet the relevant diagnostic criteria, the positive predictive value of toxoplasmosis can reach 90% [37, 38]. It should be noted, however, that cerebrospinal fluid Polymerase Chain Reaction (PCR)-negative status cannot rule out cerebral toxoplasmosis. Moreover, lumbar puncture should be avoided for patients with brain edema [39]. Other routine laboratory tests have shown that blood lactate dehydrogenase is significantly elevated in cerebral toxoplasmosis [40]. The authors of these studies suggested conducting a trial anti-toxoplasma treatment in such cases, especially for patients with low CD4^+^T lymphocytes, suspected cerebral toxoplasmosis, seropositivity for anti-toxoplasma IgG antibody, and lack of preventive measures, as efficacy is usually consistent with the diagnosis. Alternatively, when anti-toxoplasma treatment is ineffective and the diagnosis is in doubt, confirmation can be achieved by brain biopsy, although in most cases, biopsy sampling is limited by invasiveness and risk of complications.

### 3.2 Treatment and Prognosis

Treatments for AIDS-related cerebral toxoplasmosis include antibacterial and antiretroviral therapies. The most common treatment is combination therapy with pyrimidine plus the anti-toxoplasmosis agent sulfadiazine, usually for six weeks [41, 42]. Thereafter, secondary prevention and/or maintenance ART treatment should be conducted until the CD4^+^T lymphocyte count exceeds 200/µL for more than three months to prevent the reactivation of infection in latent lesions. Most clinicians also recommend steroids when the lesion has obvious brain edema [43, 44]. For patients with neurological symptoms and intracranial masses, especially those with a history of immune deficiency, pyrimidine and sulfadiazine should be used for empirical treatment [45].

Antiretroviral therapy should be initiated as soon as possible to promote immune recovery [46, 47]. Compared to mycobacterium and cryptococcus infections, immune reconstitution inflammatory syndrome caused by *T. gondii* infection will impede immune recovery. Although there is no accepted optimal time of ART for *T. gondii*-infected AIDS patients, it is usually conducted after 2 weeks of anti-toxoplasma treatment, although early antiretroviral therapy is usually effective, and a two-week delay may be unnecessary [48]. If ART is stopped, the infection may relapse with complications such as epilepsy and disturbance of consciousness. Continuous ART can reduce the risk of repeated infection [49].

### 3.3 Prevention and Prospect

The focus of infection prevention is to limit the routes of transmission based on knowledge of *T. gondii* biology, epidemiology, and ecology [50]. The standard guidelines for the prevention of cerebral toxoplasmosis suggest that all HIV-infected patients should be regularly tested for serological evidence of *T. gondii* infection, as well as immediately following potential exposure. Patients with CD4^+^T lymphocyte counts in peripheral blood less than 100/µL, and *T. gondii* seropositive patients should practice primary prevention. However, patients receiving ART with CD4^+^T cell lymphocyte count greater than 200/µL for more than 3 months can safely stop primary prevention, and patients sustaining this count for more than 6 months can stop maintenance treatment [51, 52]. Additionally, AIDS patients should avoid going to areas where toxoplasmosis is prevalent, avoid eating raw or undercooked meat, and wash their hands thoroughly after contacting cat litter or soil. Only through comprehensive clinical treatment and prevention can the incidence and mortality of cerebral toxoplasmosis be reduced [53, 54]. Although preventive measures are instituted routinely, *T. gondii* infection has not been completely eliminated [55].

In the past few decades, great progress has been made in the development of new drugs for retroviral, bacterial, and parasitic infections. Parasite-targeted therapy forms the foundation for the development of new anti-Toxoplasma drugs. Encouraging results have been reported for some existing drugs, and several new compounds with anti-toxoplasma indications have been identified [56]. Vaccination can also be used to prevent or eliminate parasitic infections (*e.g.*, *Plasmodium falciparum* malaria), suggesting the potential for developing safe and effective *T. gondii* vaccines. At present, several strategies for developing *T. gondii* vaccine are being explored, such as live attenuated strains, nanoparticles, and exosomes [57, 58]. Although significant progress has been made in vaccine research, no vaccine has been approved for human clinical trials. The major barriers to vaccine development are the complex life cycle and different antigen development stages of *T. gondii*, which trigger distinct immune responses. Therefore, the most promising research path is unknown. Nonetheless, there is general confidence that a vaccine will be developed to prevent cerebral toxoplasmosis.

## CONCLUSION

The accurate diagnosis of AIDS-related cerebral toxoplasmosis frequently requires the integration of numerous clinical symptoms and signs, multimodal neuroimaging manifestations, and serology. Current treatments provide satisfactory results in most cases if the infection is promptly diagnosed and treated. However, there are still cases of poor outcomes despite advances in imaging detection, prevention, and treatment, including ART. In these cases, new drugs and vaccines are required.

## LIST OF ABBREVIATIONS

CT: Computed Tomography
MRI: Magnetic Resonance Imaging
PET: Positron emission tomography
T1WI: T1-Weighted Images
T2WI: T2-Weighted Images
